# Lessons learned from successful autologous gastrointestinal reconstruction in patients with intestinal failure: a case series

**DOI:** 10.1186/s12893-021-01075-9

**Published:** 2021-02-04

**Authors:** Kiarash Ashrafzadeh, Mojtaba Shafiekhani, Nazanin Azadeh, Maryam Esmaeili, Hamed Nikoupour

**Affiliations:** 1 Shiraz Organ Transplant Center, Abu-Ali Sina Hospital, Shiraz University of Medical Sciences, Shiraz, Iran; 2 Shiraz Transplant Research Center, Shiraz University of Medical Sciences, Shiraz, Iran; 3 Department of Clinical Pharmacy, Faculty of Pharmacy, Shiraz University of Medical Sciences, Shiraz, Iran

**Keywords:** Autologous gastrointestinal reconstruction, Enterocutaneous fistula, Intestinal failure, Intestinal rehabilitation unit

## Abstract

**Background:**

Intestinal failure (IF) is a rare but severe form of organ failure. The condition is defined as body’s inability to absorb adequate fluids, macronutrients and minerals for growth and development, so that intravenous supplementation is necessary. A broad spectrum of diseases, trauma and complications of surgery might eventually end up with intestinal failure. Nowadays, intestinal failure patients are preferably cared for in intestinal rehabilitation units (IRU). Autologous gastrointestinal reconstruction (AGIR) refers to non-transplant operative management of IF patients designed to improve enteral tolerance and gut absorptive capacity.

**Case presentation:**

Herein we present five cases with complications of surgeries due to peptic ulcer bleeding, blunt abdominal trauma, obesity and gastric tumor. The surgeries were complicated by anastomotic leak, peritonitis and fistula formation. By adopting multidisciplinary decisions and special care for each complication, all the five patients were successfully managed and discharged.

**Discussion and conclusions:**

As presented, re-anastomosis in presence of abdominal contamination will probably fail. In patients with intestinal failure, PN should start as soon as possible to increase the success rate of future surgeries and prevent potential need for intestinal transplantation. We suggest referring patients with complicated outcomes of gastrointestinal surgeries to the IRUs to reduce morbidity and mortality.

## Background

In 1981 Fleming et al., defined intestinal failure (IF) as a reduction in the gut’s function to absorb the minimal necessary amount of food to meet the adequate energy requirements [1]. Later, the definition was revised and the body’s dependence on intravenous supplementation to maintain health and growth was included in the definition of IF [2]. A wide variety of conditions compromising the small bowel’s function may eventually end up with IF. The major etiologies are reported to be iatrogenic as a complication of surgery, malabsorption syndromes, vascular events, trauma, intra-abdominal malignancies, congenital causes and motility disorders [3]. The mentioned conditions might result in IF with various mechanisms including short bowel syndrome (SBS), enterocutaneous fistula, mechanical obstruction, extensive mucosal disease and intestinal dysmotility [4]. One major cause of IF is surgical resection of the small intestine. Starting as soon as 24 to 48 h following an extensive resection of the small bowel, the remaining segments will undergo adaptive changes for efficient absorption. The changes include increasing villi height and crypt depth, enterocyte and smooth muscle hyperplasia with consequent small intestine hypertrophy. The adaptive changes will continue for months [5, 6].

The aim of management of IF is to reduce complications, treat or alleviate the underlying disease, provide rational and proper intravenous supplementation and achieve a desirable quality of life for the patients [7]. Due to the chronic nature of underlying conditions of IF, possibility of numerous complications and long-term treatment, in most centers a multidisciplinary management has been adopted. The multidisciplinary team (MDT) includes surgeons, pharmacists, nutritionists, gastrointestinal specialists, trained nurses and various other experts. They provide a management plan to shorten the hospital admission period, reduce the need for intestinal transplantation and advocate home parenteral nutrition (PN) [8].

Autologous gastrointestinal repair (AGIR) refers to a variety of surgical techniques adopted by MDT for IF patients with SBS aimed to encourage the remnant small bowel’s adaptation process and consequently provide a sufficient absorptive mucosa. Surgeons may repair fistulas, take down stomas, elongate the small intestine and resect the diseased segments as various techniques of AGIR. By the help of AGIR, a greater mucosal area will be exposed to enteral material enhancing the adaptation process [9]. Furthermore; AGIR helps lower the risk of bacterial overgrowth by reducing segmental intestinal dilatation [10].

The intestinal rehabilitation unit (IRU) of the Abu Ali Sina hospital in Shiraz, Iran is a unique center in the Middle East which has been a place for management of patients with IF since 2018 with outstanding outcomes. The hospital is the biggest center of solid organ transplantation in Asia and is affiliated to Shiraz University of Medical Sciences, Shiraz, Iran. Currently, the IRU has 14 beds with an average annual admission of 50 patients with IF. The MDT consists of hepatobiliary surgeons as the head of the team, a clinical pharmacist assigned for designing and management of pharmacotherapy and PN, wound care specialists, expert nurses and registered dietitians. In the current article we first briefly review successful managements of five complex IF cases referred to our center, their history and outcomes. Then, we provide surgical tips to prevent the primary mismanagements of these patients as follows.

## Case presentation

### Case 1

#### History and presentation

Previously healthy 45-year-old gentleman presented to the emergency department (ED) complaining of hematemesis. After initial resuscitation with fluids, while hemodynamically stable, the patient underwent diagnostic and therapeutic upper gastrointestinal endoscopy, during which a bleeding duodenal ulcer was seen. The surgery team was called for an emergent laparotomy when the procedure was not successful in controlling the bleeding site. After hemostasis and doudenotomy, he was transferred to the ward. Few days later, he developed peritonitis due to duodenal suture leak.The patient underwent a secondary laparotomy for inserting a draining tube and was then transferred to our center for further management.

#### Management

**Fig. 1:**
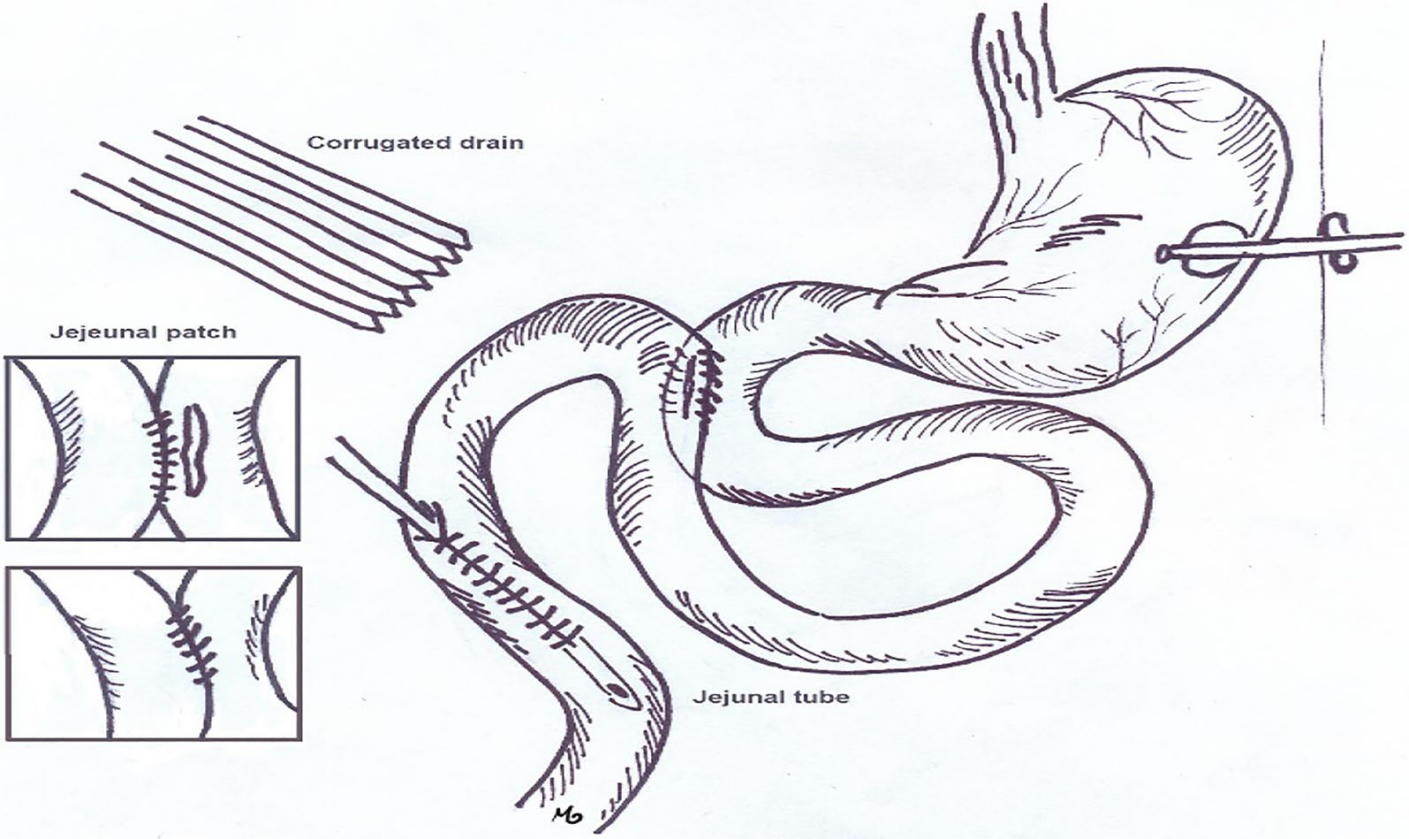
Scheme depicting the interventions done for patient with duodenal suture leak. Corrugate drain, gastrostomy and jejunostomy tubes were inserted and the anastomosis site was repaired with jejunal serosal patch technique

As it had passed less than 10 days from the first surgery, we decided to repair the anastomosis site with jejunal serosal patch technique and inserted a gastrostomy and jejunostomy tube. Besides; a corrugated drain was inserted in the abdominal cavity (Fig. 1). On the second post-operation day, we noticed bile leakage via the corrugated drain indicating failure of the jejunal serosal patch and ongoing bile leakage from the anastomotic site to the abdominal cavity. The daily leakage was approximately 500 cc in the early post-operative days and gradually vanished in 4 weeks. During these days, the patient received nutrition via jejunostomy tube as well as broad spectrum antibiotic therapy. At the end of the 4th week, the patient tolerated oral diet and no drainage from the corrugated drain was recorded. At this time the gastrostrostomy, corrugated drain and jejunostomy tubes were respectively removed and the patient was discharged in a relatively good state of health.

### Case 2

#### History and presentation

**Fig. 2:**
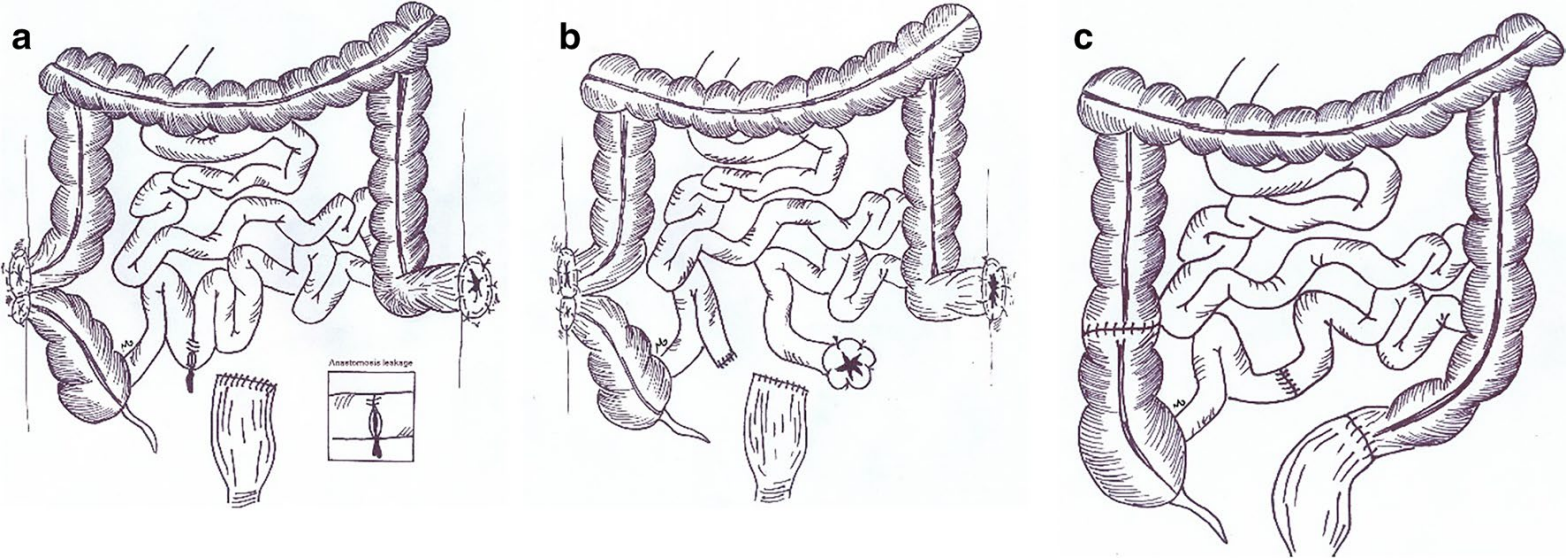
The scheme depicting the prior surgical interventions before referral. The patient was transferred to our center with a loop colostomy of ascending colon, sigmoid colostomy with Hartman pouch and leakage from ileal anastomosis site (**a**). Scheme depicting ileal anastomosis takedown with creation of an ileostomy as the first management plan in our center (**b**). In The last operation, the colostomies were reversed and a side to side ileal anastomosis was created at the site of previous ileostomy (**c**)

A 30 year old gentleman was admitted in the ER following a car-patient accident. The patient was immediately examined by a general surgeon. The surgeon noticed abdominal guarding with generalized rebound tenderness and arranged an emergent operation for him. During the operation he underwent resection of extensively injured small bowel segments. A loop colostomy of ascending colon, sigmoid colostomy and Hartman pouch were created as well. Within 10 days, he underwent two further laparotomies due to anastomosis site leakage. The patient was transferred to our center with ongoing ileal anastomosis site leakage, peritonitis, two stomas (ascending colon loop colostomy and sigmoid colostomy) with an open abdomen (Fig. 2a).

#### Management

We transferred the patient to the OR and took down the ileal anastomosis and inserted an ileostomy 100 cm down to ligament of Treitz (Fig. 2b). A vacuum dressing was applied on the open abdomen and PN was started for the patient. After weaning the patient from the PN, he was discharged.

A final surgery was done 6 months later to regain gastrointestinal autonomy. In the procedure, a side to side small bowel anastomosis was done. Further, the sigmoid and ascending colon loop colostomies were reversed (Fig. 2c).

### Case 3

#### History and presentation

**Fig. 3:**
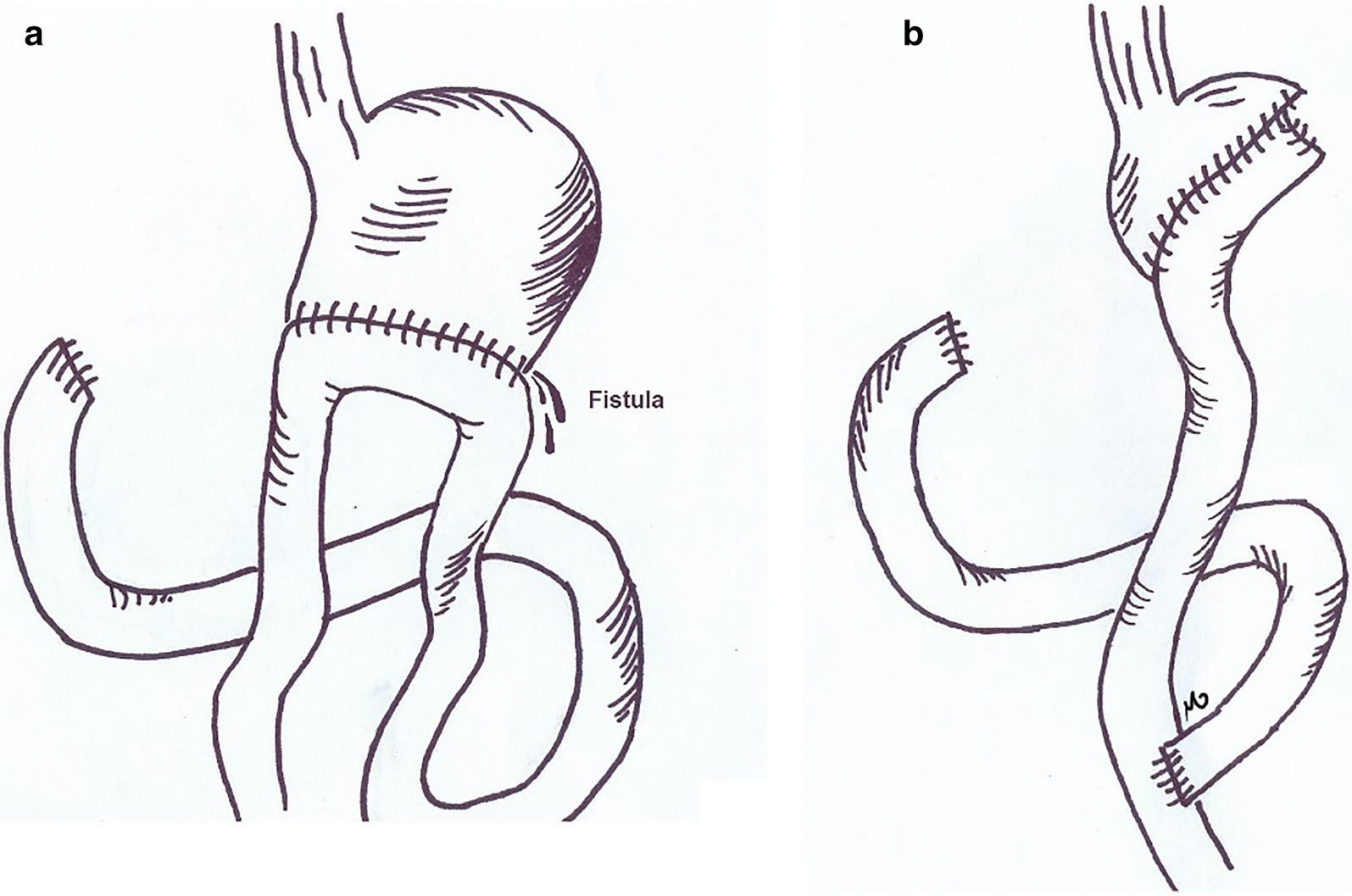
The patient was admitted after the Bilroth II procedure with a gastrocutaneous fistula and gastrojejunostomy leak (**a**). In the final surgery no tumoral recurrence was detected and after fistulectomy, Roux-en-Y procedure was done for her (**b**)

A 40 year old lady had undergone hemigastrectomy and Billroth I operation with diagnosis of gastric neuroendocrine tumor (NET), few days later, due to anastomostic leak, the patient underwent a second laparotomy in which the surgeons took down the anastomosis, closed the duodenal stump and created a loop gastrojejunostomy (Bilroth II). Later post-operation assessment was indicative of a further duodenal stump blow out and leakage from the site of gastrojejunostomy as well as a gastrocutaneous fistula (Fig. 3a). The patient was transferred to our center with peritonitis and subsequent sepsis.

#### Management

We took her to the OR for abdominal lavage in six sessions. The patient was admitted in ward with a vacuum dressing and daily abdominal fluid drainage of an approximate 1 L. With the same ongoing management, she recovered from sepsis and was put on PN. At the end of the 5th week, the daily abdominal fluid drainage had reached zero, patient had tolerated oral nutrition and her wound was healing with granulation tissue. Soon, she was discharged with close follow up. A year later, she was admitted with a high output enterocutaneous fistula. No tumor recurrence was detected in workups and the patient was scheduled for exploratory laparotomy. In the OR, we noticed a stricture in the efferent loop of gastrojejunostomy resulting in reopening of the fistula. As no tumor recurrence was observed, the patient underwent subtotal gastrectomy with Roux-en-Y anastomosis and two layer closure of duodenal stump (Fig. 3b).

### Case 4

#### History and presentation

A 13 year old young man was transferred to OR after blunt trauma to abdomen. During the operation, hematoma of small intestine mesentery was observed and the surgeon decided to close the abdomen with no action. A week later, the patient underwent re-operation with diagnosis of peritonitis. In the OR the surgeon noticed a necrosed and perforated segment of small intestine measuring 30 cm in length and 5 cm distal to ligament of Treitz. Meanwhile; the abdomen was filled with fecal material. The necrosed small bowel segment was resected and jejunal anastomosis was created. In the 5th post-operation day, the patient developed anastomotic leak and peritonitis and was referred to our center.

#### Management

**Fig. 4:**
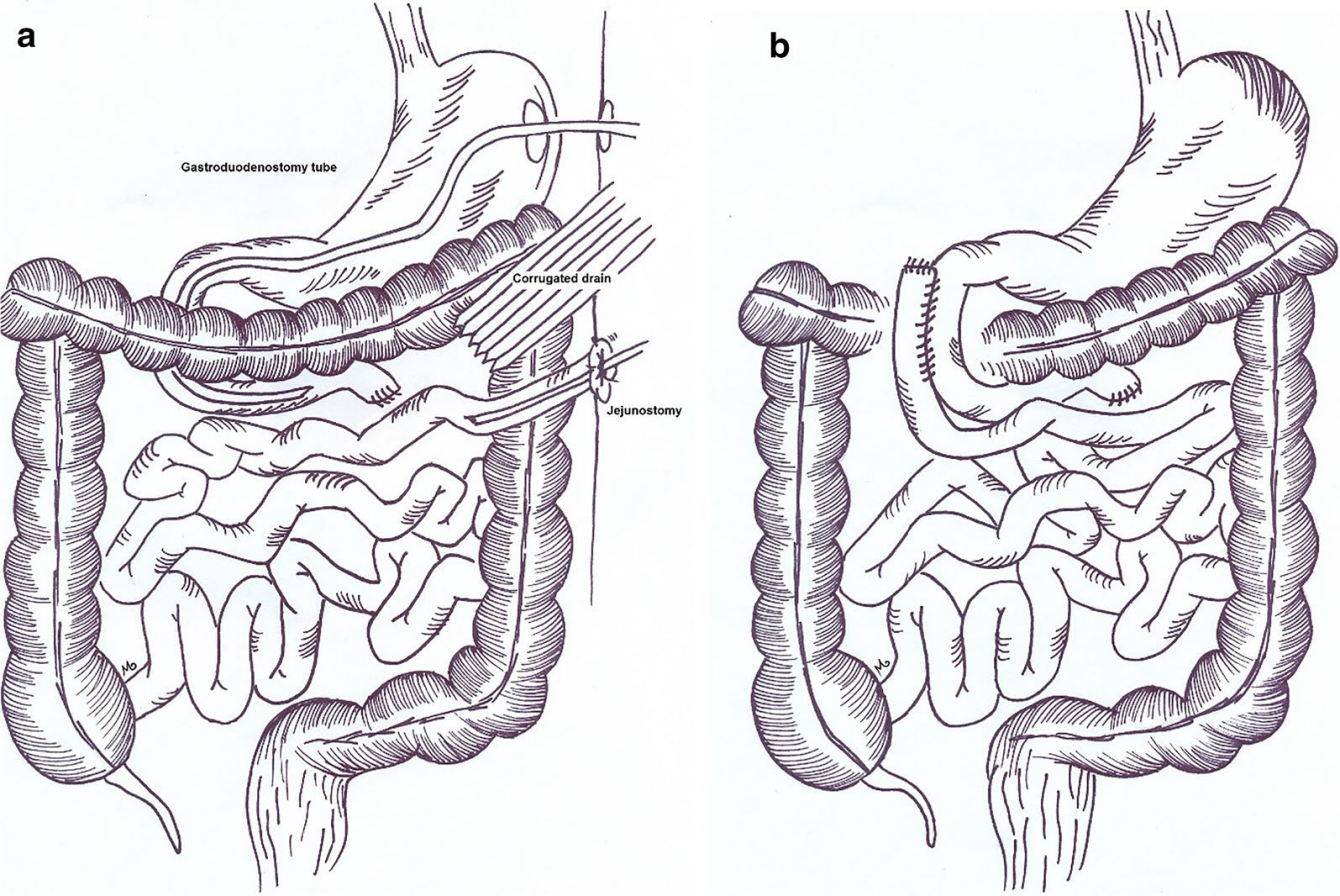
The jejunal anastomosis was took down and corrugated drain, gastrostomy and jejunostomy were inserted (**a**). In the final surgery a side-to-side jejunodoudenal anastomosis was done (**b**)

We took him to the OR and observed the jejunal stump dehiscence, for which we placed a corrugated drain (Fig. 4a). Abdominal washout was done and a gastrojejunostomy tube was inserted; as well as a jejunostomy tube. During the following weeks, the patient was admitted in the IRU and received nutrition via jejunostomy tube while the fistula was healing. In the 30th post-operation day, he had no recorded duodenal fluid drained in the tube. The tube was removed and he was discharged. Six months later, during an elective surgery we created a side to side duodenum to jejunum anastomosis for him with no complication (Fig. 4b).

### Case 5

#### History and presentation

**Fig. 5:**
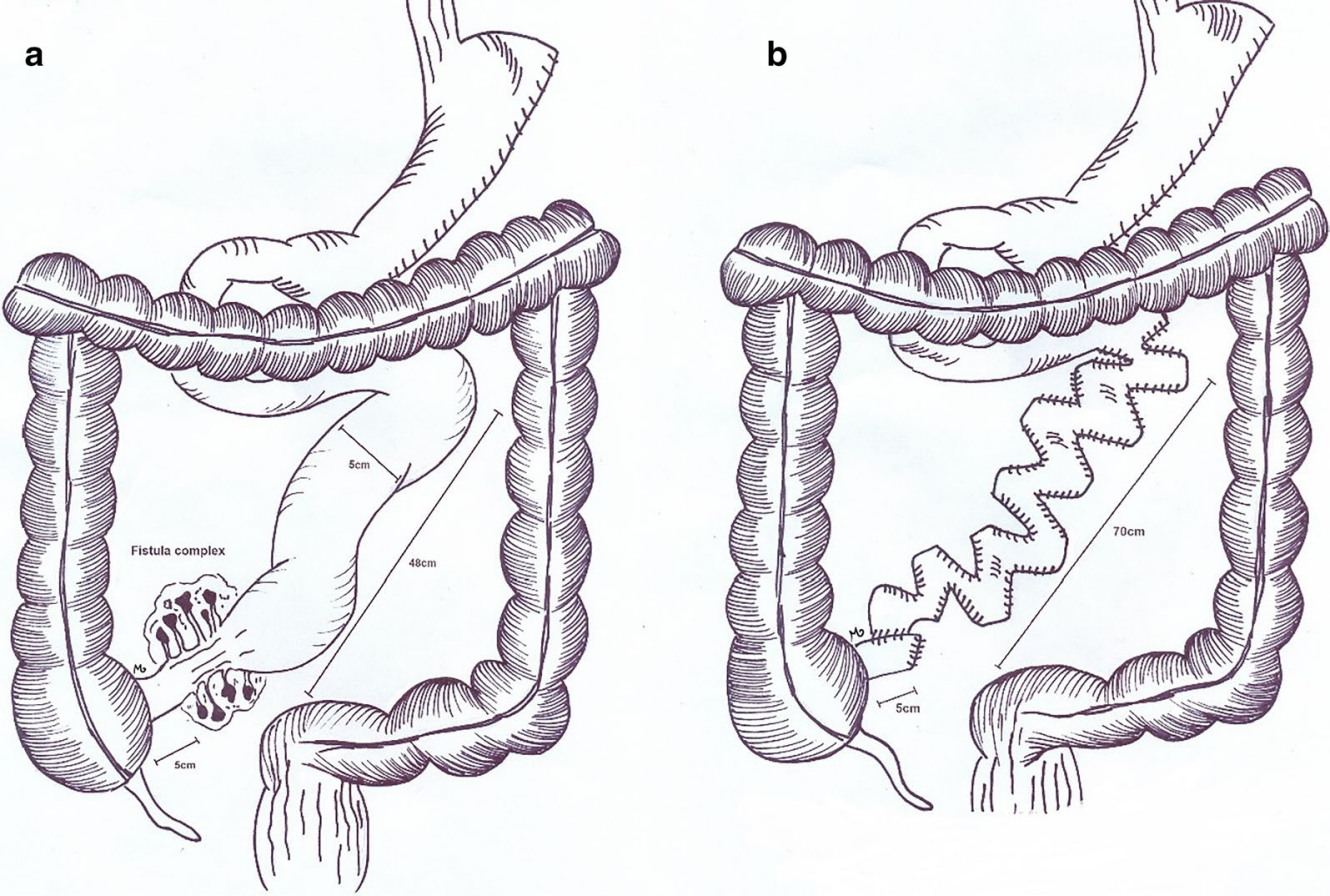
The patient was referred to our center with an extensively resected small intestine and a large fistula island (**a**). The small bowel was lengthen using the STEP technique (**b**)

A 37 year old morbid obese male patient underwent Sleeve gastrectomy complicated by post-operation portal vein thrombosis (PVT) and subsequent intestinal necrosis. The patient underwent six more operations for resection and anastomosis of the gangrened small bowel parts. The operations were complicated by a large fistula island superior to the umbilicus with 1000 cc daily output (Fig. 5a). It was estimated the remnant of small bowel would be 50 cm length. The patient was hospitalized in the primary hospital for a month and was referred to our center for further management.

#### Management

As soon as the patient got admitted in our ward, due to malnutrition status, we started oral nutrition as well as PN. By the 6th month, the patient was in a desirable state to undergo a reconstructive surgery. The intra-operative findings were as following; 48 cm of small bowel was preserved distal to ligament of Treitz. The bowel remnant was 5 cm in diameter and a complex fistula was found 5 cm proximal to the ileocecal valve. Due to PVT and formation of collateral veins, the patient bled 3.5 L during the operation. At the end of the procedure we had increased the intestinal length to 75 cm by serial transverse enteroplasty technique (STEP) (Fig. 5b). In the 15th post-operation day, we noticed a low output enterocutaneous fistula. For the following 2 weeks oral nutrition was withheld for the patient and he was put on PN, during this period the fistula’s output gradually declined to 10 cc per day. As soon as the time the patient tolerated oral nutrition, he was discharged in a fairly good condition.

## Discussion and conclusion

IF is a complex clinical problem requiring coordinated multi-disciplinary care. With proper surgical and medical decisions the risk of chronic IF could be lowered [2]. Due to severity of underlying conditions, patients with IF need a specialized management medical and surgical plan which is usually provided in IRU [8].

Herein, we have provided five cases of IF patients who were referred and successfully treated in our center as examples. The first presented case had developed duodenal leak and peritonitis following emergent duodenotomy. Anastomotic leak is a devastating complication of gastrointestinal surgeries. In cases of delayed diagnosis, the leaked intraluminal materials expose the abdominal cavity to a large number of bacteria. When peritonitis occurs, emergent laparotomy and abdominal washout is crucial as well as broad-spectrum antibiotics and fluid resuscitation. Re-anastomosis is not recommended in presence of peritonitis, as the risk of further leakage is increased [11]. One may consider pyloric exclusion in such case, but the procedure necessitates creation of a gastrojejunostomy which could end up with another leakage in this patient with intra-abdominal contamination and unstable state. We recommend the least possible surgical procedures in presence of abdominal contamination. As the patient needed to stay nil by mouth (NPO) for a relative long period of time he needed a nutritional root to be created. In our country, home PN is not practiced; besides prolonged PN might result in several side effects including intestinal failure liver associated diseases, hypoglycemia, electrolyte imbalance and catheter infections [12]. Considering these facts, we decided to insert a jejunostomy tube instead, so patient’s costs and length of hospital stay would be reduced as well. Furthermore; we inserted a corrugated tube in the duodenum for drainage of secretions. However the applied jejunal serosal patch procedure failed in presence of abdominal contamination, a second leak by re-anastomosis was not imposed on the patient.

The 2nd case was a young man with peritonitis and ongoing anastomotic leak from ileum. When facing anastomotic leak, antibiotic therapy, percutaneous or surgical drainage, and reoperation with anastomosis take down or diversion are the treatment options. Surgeons will adopt one approach depending on severity of peritonitis and presence of abscesses. Salvage of anastomosis will be considered in limited local peritonitis or abscess formation. Usually when reoperation is needed to take down the anastomosis, a stoma will be created [13, 14]. In this case we avoided further ileal resection and anastomosis due to high risk of leak in presence of diffuse peritonitis. Therefore; we decided to take down the anastomosis and create an ileostomy. It is wisely advised to preserve the ileocecal valve in cases of multiple resections of small bowel. Presence of the ileocecal valve slows down the ileal transit time allowing for more absorption. Besides; resection of the valve will colonize the small intestine with colon bacteria resulting in diarrhea [15].

The 3rd case was a patient with anastomosis leak from the Billroth I procedure who later underwent Bilroth II. Bilroth I procedure is a simple and more physiologic technique; however it is accompanied by more tension at the anastomotic site. It is suggested that surgeons consider other surgical techniques rather than Biltoth I when a tension free anastomosis is not possible [16]. To prevent the same mismanagement as the one applied for this patient, we suggest no further anastomoses in unstable patients with ongoing leak. In this case, our management plan was daily washout of the abdomen until confinement of leak sites and cessation of its intra-abdominal spread. Fortunately, this strategy saved the patient’s life. The last note to be kept in mind is to consider local recurrence of tumor or distal stenosis in reopened fistulas [17].

The 4th case was a blunt trauma induced mesenteric hematoma which led to ischemia and necrosis of small bowel. Mesenteric hematomas without active bleeding can be conservatively managed [18]. We suggest close observation with abdominal examination, imaging and a second look via laparotomy to prevent such disastrous outcome. As previously stated, in cases of abdominal contamination and hemodynamic instability, anastomosis is not a wise decision and there is a high probability of failure and leak, especially near ligament of Treitz. In this patient we created a jejunostomy due to predicted long time of reconstructive surgery. The final surgery was done 6 months later, during this period the patient gained weight and received nutritional support via jejunostomy. In the final surgery we used hand sewn side-to-side anastomosis rather than the end-to-end technique, as the previous studies have shown fewer incidence of leakage and other complications in the prior anastomosis technique [19].

The final case was a patient with post-Sleeve gastrectomy portal vein thrombosis and bowel gangrene. The number of bariatric surgeries are rising as a result of increasing obesity, surgeons should be prepared for potential complications. In cases of post-operation complications and bowel compromise patients should be referred to centers with IRUs. In this patient, insisting on several operations in a short period of time without proper healing or resolution of infection led to worsening of situation and formation of fistula island. In the OR, decision on surgical technique of bowel reconstruction is made after resection of the fistula complex and depends on diameter and length of the bowel remnant. In this case the bowel remnant was short in length (48 cm) and we used the serial transverse enteroplasty (STEP) technique to lengthen the small intestine to 75 cm. The STEP procedure is done by creating zigzag patterns along the intestine to increase its length and consequently; the absorptive mucosa in patients with SBS [20]. Numerous reports of STEP have yielded a relatively favorable outcome in the pediatric field; while fewer adult reports are available. The STEP procedure has shown promising results in alleviating malabsorption and reducing the need to intestinal transplantation [21]. The procedure is widely accepted due to its non-complicated technique and minimal compromise to mesenteric vasculature. By decreasing the diameter of dilated bowel loops, STEP has been shown to reduce the risk of bacterial overgrowth [21].

Here we presented five patients who were referred to our center with overviewing their primary mismanagements, healing processes and preferred surgical interventions. In cases of complicated gut injury we suggest eminent referring of the patients to IRUs to reduce mortality and morbidity. Unavailability of home PN in some countries has led to inappropriate surgical decisions such as bowel anastomosis in presence of abdominal contamination and re-anastomosis after previous leakage. The unfavorable decisions increase morbidity and hospital stay. Time of the second operation in patients with abdominal contamination should be carefully decided. In our center we schedule AGIR operations either before the 10th post-operation day when abdominal contamination has not extensively spread and patients have not turned malnourished or later than the 120th day, when patients have gained weight by the help of PN and better tolerate surgeries. We operate the open abdomen cases either after 2 months or 6 months. The latter is adopted for the patients with severe abdominal contamination. Classically, we practice hand sewn 2-layered anastomoses with prolene or polydioxanone (PDS) stitches.

Due to complexity and multifactorial features of IF, its management must be individualized according to patients’ clinical status, anatomy of intestine and assessment the risk of following complications. In cases of anastomotic leak or fistula formation, it is advised that surgeons avoid quick decisions and follow guidelines to lessen morbidity and mortality rates. We suggest no further non-urgent surgeries in complicated cases of bowel compromise and referring patients to IRUs for appropriate management.

## Data Availability

Not applicable.

## Abbreviations

AGIR: Autologous gastrointestinal reconstruction
ED: Emergency department
IF: Intestinal failure
IRU: Intestinal rehabilitation unit
MDT: Multidisciplinary team
NLM: Nil by mouth
NET: Neuroendocrine tumor
OR: Operation room
PN: Parenteral nutrition
PVT: Portal vein thrombosis
STEP: Serial transverse enteroplasty
